# Disrupted topological properties of functional networks in epileptic children with generalized tonic‐clonic seizures

**DOI:** 10.1002/brb3.1890

**Published:** 2020-10-24

**Authors:** Yongxin Li, Qian Chen, Wenhua Huang

**Affiliations:** ^1^ Formula‐pattern Research Center School of Traditional Chinese Medicine Jinan University Guangzhou China; ^2^ Department of Pediatric Neurosurgery Shenzhen Children Hospital Shenzhen China; ^3^ Guangdong Provincial Key Laboratory of Medical Biomechanics School of Basic Medical Sciences Southern Medical University Guangzhou China

**Keywords:** epileptic children, functional connectivity network, generalized tonic‐clonic seizure, graph theory, resting‐state fMRI, topological organization

## Abstract

**Introduction:**

Generalized tonic‐clonic seizure (GTCS) is a condition that is characterized by generalized spike‐wave discharge in bilateral cerebral hemispheres during the seizure. Although previous neuroimaging studies revealed functional abnormalities in the brain activities of children with GTCS, the topological alterations in whole‐brain networks remain poorly understood.

**Methods:**

The present study used graph theory to investigate the topological organization of functional networks in 13 GTCS children and 30 age‐matched healthy controls.

**Results:**

We found that both groups exhibited a small‐world topology of the functional network. However, children with GTCS showed a significant decrease in nodal local efficiency and clustering coefficient in some key nodes compared with the controls. The connections within the default mode network (DMN) were decreased significantly, and the internetwork connections were increased significantly. The altered topological properties may be an effect of chronic epilepsy. As a result, the optimal topological organization of the functional network was disrupted in the patient group. Notably, clustering coefficient and nodal local efficiency in the bilateral temporal pole of the middle temporal gyrus negatively correlated with the epilepsy duration.

**Conclusion:**

These results suggest that the bilateral temporal pole plays an important role in reflecting the effect of chronic epilepsy on the topological properties in GTCS children. The present study demonstrated a disrupted topological organization in children with GTCS. These findings provide new insight into our understanding of this disorder.

## Graphical abstract

(a) Children with GTCS exhibited a small‐world topology of their functional network; (b) Detected significant decrease of regional topological organization in children with GTCS; (c) Network metrics in bilateral temporal pole of MTG showed negative correlations with the epilepsy duration.

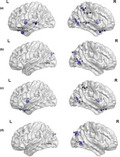

## 1 INTRODUCTION

Epilepsy is one of the most common, serious, and chronic neurological disorders, and it affects 65 million people worldwide (Thurman et al., 2011). Generalized tonic‐clonic seizure (GTCS) generally produces bilateral, convulsive tonic, and clonic muscle contractions (Wang et al., 2018). Previous studies indicated that GTCS needed more medical attention in clinical practice (Engel & International League Against Epilepsy, 2001; Kenyon et al., 2014). People with this type of epilepsy show cognitive deficits in memory, attention, and executive function (Hommet et al., 2006). Their quality of life is also affected by epilepsy, especially in children (Fayed et al., 2015). Considerable efforts were made recently to examine the mechanisms of epilepsy, and some important achievements were obtained (Bernhardt et al., 2009; Ji et al., 2014; Kenyon et al., 2014; Thurman et al., 2011). However, the neural mechanisms of GTCS remain poorly understood and have attracted much attention in clinical research (Liu et al., 2017).

Neuroimaging techniques were developed recently, and these techniques were used to examine the underlying neural mechanisms of epilepsy and the intervention effects of clinical treatment (Li et al., 2017; Reyes et al., 2016; Wang et al., 2017; Zhang et al., 2014). Functional magnetic resonance imaging (fMRI) is an extensively used method in clinical research because the method is efficient and noninvasive. Numerous neuroimaging studies in adults with GTCS found significant changes in brain structure (Ciumas & Savic, 2006; Huang et al., 2011; Li et al., 2010; Zhou et al., 2015) and function (Parsons et al., 2020; Wang et al., 2014; Zhong et al., 2011) compared with normal controls. For example, a neuroimaging study of gray matter volume of the hippocampus demonstrated a significant reduction and correlation with some aspects of cognitive impairment in adults with GTCS (Zhou et al., 2015). Whole‐brain structural‐imaging studies have also detected significant volume decreases in the bilateral frontal lobe, thalamus, insula, and cerebellum of adults with GTCS (Huang et al., 2011). Impairment of the thalamocortical structural network may be the main expression of epilepsy effects in the structural domain. In the functional domain, resting‐state fMRI is the generally used approach to evaluate regional interactions (Bullmore, 2012). Zhong et al. (2011) calculated regional homogeneity to measure the synchronization of spontaneous signal oscillation within spatially neighboring voxels. Adults with GTCS showed bilateral and symmetrical alterations in regional homogeneity in cortical and subcortical regions. Another resting‐state fMRI study in adults with GTCS calculated the amplitude of low‐frequency fluctuations and detected significant brain activity differences in the thalamus and prefrontal cortex (Wang et al., 2014). Decreased spontaneous activity was primarily found within the default mode network (DMN) (Parsons et al., 2020). Our recent neuroimaging studies also found that children with GTCS showed significantly reduced gray matter volume and increased spontaneous activity in temporal lobe, hippocampus, thalamus, and other deep nuclei (Wang et al., 2018). Together, these neuroimaging findings suggest that functional and structural changes occur in the thalamus, temporal lobe, and DMN after epilepsy.

Analyses of structural and functional connectomics have become a recent research focus in epilepsy. The human brain is a complex network. The function of the network depends on connectivity patterns. Applying this method to epilepsy provides profound insight into the underlying reorganization of the effects of epilepsy on the topological properties of the brain. For example, adults with GTCS show functional reorganization of the DMN and dorsal attention network (Wang et al., 2011). Significant increases in voxel‐mirrored homotopic connectivity are also found in the bilateral anterior cingulate and medial prefrontal gyrus of adults with GTCS (Yang et al., 2014). Dynamic functional connectivity was evaluated using resting‐state fMRI, and adults with GTCS demonstrated state‐specific disruptions of functional network connectivity (Liu et al., 2017). Aberrant interhemispheric functional and anatomical connectivity were also detected in adults with GTCS (Ji et al., 2014). Although the above previous studies advanced our understanding of brain aberrant connections in patients with GTCS, the topological features of the whole‐brain network have lagged behind in this disease. Most previous studies focused on regional connectivity changes. The human brain is a complex information processing system in which different brain regions are coordinated as a functional network (Xia & He, 2017). This view motivated us to study brains with neurological disorders from a whole‐brain complex network perspective based on graph theory analysis.

Graph theory is a mathematical method that is used to quantify network topology (Engel et al., 2013). Graph theory envisage the brain in terms of the connectome, such as a system of nodes (brain anatomical regions) and edges (structural or functional connections) (Bullmore & Sporns, 2009). Its unified framework was used to investigate the network topology of the human brain. The network topological properties are quantified as small‐world property, global/local efficiency, and clustering coefficient. The local efficiency is a network parameter that reflects the short‐range connections between neighboring regions. The clustering coefficient is a network parameter that provides information about the level of local neighborhood clustering within a graph. The topological parameter may be used to express how close the neighbors nodes are connected to themselves (Watts & Strogatz, 1998). Application of the graph theory in human brains detected that the normal human brain network exhibits a “small‐world” organization (Bassett & Bullmore, 2006). This network organization exhibits the following main features: high local clustering of nodes and minimal path length between the nodes (Watts & Strogatz, 1998). The human brain is a balanced combination of segregated and integrated brain information processing. More efficient rates of information processing and learning are allowed with this brain organization. Because the human brain network is described quantitatively by small‐world characteristics, the graph‐theoretical method has been widely applied to assess the organization of brain functional and structural networks in healthy individuals and patient populations (Cao et al., 2013; Helena et al., 2018; Suo et al., 2015; Zhao et al., 2015). A previous neuroscience study indicated that epilepsy was a disease of structurally and/or functionally aberrant connections between neurons (Engel et al., 2013). The use of the graph theory approach will help our understanding of the neural mechanisms underlying epilepsy (Bernhardt et al., 2015; Onias et al., 2014). Few neuroimaging studies have used graph theory to examine the disruption of brain topological properties in adults with GTCS (Li et al., 2016; Liao et al., 2013; Song et al., 2011; Zhang et al., 2011). These previous neuroimaging studies demonstrated that graph theory provided further network‐level information about the pathology of epilepsy patients.

However, the objects of the above mentioned studies were adult patients with GTCS. One recent neuroimaging study included both children and adults with generalized epilepsy of GTCS and found that patients showed a more constrained network embedding of the thalamus and increased functional diversity in frontocentral neocortical regions (Wang et al., 2019). One recent study from our group also focused on children with GTCS and detected significant changes in brain gray matter volume and spontaneous brain activity in patients (Wang et al., 2018). Using graph theory method on the gray matter structural covariance network, children with GTCS showed significant changes in nodal betweenness locating in brain regions such as thalamus, temporal pole, and some regions of DMN (Li et al., 2020). Although specific changes of brain activity, functional connectivity and regional topology disruption of structural covariance network were detected in these previous studies of children with GTCS, the topological organization of their brain functional network is remain unclear. Graph theory analysis could provide a powerful framework for characterizing the topological architecture of the brain connectome for children with GTCS. Given the previous evidence of abnormal regional activities and gray matter volumes in widely distributed regions, together with the findings of impaired structural topological properties in children with GTCS, we hypothesized that the brain functional networks in children with GTCS would follow a small‐world organization. We also hypothesized that altered topological organization of the brain functional connectome would involve the DMN and temporal regions. These topological changes in the patient group significantly correlated with epilepsy duration. To achieve these aims, the present study examined the global and regional network topological organization in children with GTCS and compared these networks to healthy subjects. The relationship between network topological organization and epilepsy duration was further investigated.

## 2 METHODS

### 2.1 Subjects

Thirteen children with GTCS (3 female, mean age: 4.22 ± 2.43 years, age range: 0.6–10 years) were recruited. All patients were diagnosed with GTCS. The following inclusion criteria were used: (a) typical clinical symptoms of GTCS according to the current International League Against Epilepsy (ILAE) seizure type classification (Fisher et al., 2017), such as limb movement, loss of consciousness during seizures, and no partial seizures; (b) a specific pattern of electrophysiological activity on electroencephalogram, in which generalized spike‐and‐wave or poly‐spike‐wave discharges was recorded; and (c) no focal abnormality in routine structural MRI examinations. Table 1 summarizes the demographic and clinical information of all patients. Each patient was treated with at least one antiepileptic drug (valproic acid, topiramate, and/or levetiracetam) to control seizures before imaging data collection. All patients were seizure‐free for at least two days prior to MRI examination. Thirty healthy children (HC, 18 female, mean age: 5.10 ± 2.18 years, age range: 1.08–10.67 years) without neurological disorders or psychiatric illnesses were served as the controls. During the MRI scanning, participants under the age of four were sedated with 10% chloral hydrate to reduce their head movement.

**TABLE 1:** Summary of the clinical characteristics of child epilepsy patients

Patient no.	Sex	Age (years)	Age of epilepsy onset in years	Duration (years)	Antiepileptic drugs
1	M	0.67	0.04	0.63	Oxcarbazepine, Valproic acid
2	M	3.92	3.92	0.12	Lamotrigine
3	F	4.58	0.17	4.5	Oxcarbazepine, Levetiracetam
4	F	4.58	1.58	3	Oxcarbazepine, Levetiracetam
5	M	6.67	0.5	6.17	Valproic acid, Levetiracetam
6	M	10	6	4	Topiramate, Levetiracetam
7	M	5.67	5.67	0.03	Lamotrigine
8	M	3.33	1.5	1.83	Lamotrigine, Valproic acid
9	M	0.58	0.08	0.42	Oxcarbazepine
10	F	3.25	0.33	3	Topiramate, Lamotrigine
11	M	4.33	0.75	3.42	Topiramate, Levetiracetam
12	M	3.08	3	0.17	Valproic acid
13	M	4.25	0.75	3.5	Lamotrigine

Abbreviations: F, female; M, male.

Before the image data were collected, the study purpose, procedures, possible risks, and discomforts were explained to the participants and their parents. The study was approved by the Institutional Ethic Committee of Shenzhen Children's Hospital, and the parents or the guardians of all participants signed informed consent forms.

### 2.2 Data acquisition

All images were acquired using a 3T Siemens scanner (MAGNETOM Trio Tim, Siemens, Germany, 8‐channel head coil) at the Shenzhen Children's Hospital, Shenzhen, China. To reduce head movements and scanner noise, foam cushions were used to fix the head of each participant during the scan processing. Three‐dimensional structural images of T1‐weighted MPRAGE images covering the entire brain were obtained for all but one control subject: repetition time (TR) = 2,300 ms, echo time (TE) = 2.26 ms, field of view (FOV) = 200 × 256 mm^2^, a matrix = 200 × 256, 160 sagittal slices, slice thickness = 1 mm, flip angle = 8°. Resting‐state fMRI data were acquired using an echo‐planar imaging sequence with 130 volumes: TR = 2000 ms, TE = 30 ms, FOV = 220 × 220 mm^2^, matrix size = 94 × 94, slice thickness = 3 mm, flip angle = 90°, 36 interleaved axial slices covered the entire brain. During the imaging scanning, all participants were instructed to close their eyes and remain awake, and instructed not to think about anything. All participants were lying quietly.

### 2.3 Data preprocessing

Processing the processing of resting‐state fMRI data was performed using Data Processing Assistant for Resting‐State fMRI software (DPARSF, http://www.restfmri.net) (Yan & Zang, 2010), which runs on MATLAB 8.2 (Mathworks). Processing comprised the following steps: (a) The first 10 volumes of individual functional images were discarded to ensure magnetization equilibrium. (b) The remaining volumes were corrected by the acquisition time delay among different slices and realigned to the first volume to correct the head motions. All of the subjects in the present study had less than 2 mm of translation and 2° of rotation in any of the *x*‐, *y*‐, and *z*‐axes. (c) Subsequently, each subject's high‐resolution anatomical image was coregistered to the mean functional images by rigid body transformation. (d) The transformed structural images were then segmented in to gray matter, white matter, cerebrospinal fluid by using a unified segmentation algorithm. A customized template was created based on the segmented images by using the Diffeomorphic Anatomical Registration Through Exponentiated Lie Algebra method. (e) The motion corrected functional images of each subject were spatially normalized to the Montreal Neurological Institute space by using the customized template and resampled to a 3 mm isotropic voxel. (f) The resulting images were spatially smoothed with a Gaussian filter of 6 mm full‐width half‐maximum kernel. (g) The smoothed images were further processed via temporal band‐pass filtering (0.01–0.08 Hz) and linear detrending.

To minimize the potential effects of head motion on subsequent graph theory analyses, mean framewise displacement values were also calculated during the realignment steps and compared between the two groups. For each subject, the mean framewise displacement values were the across translational and rotational directions of scan‐to‐scan deviations between two images (Power et al., 2012). No participant was excluded, and the mean framewise displacement value exceeded 0.5 mm. The framewise displacement was not significantly different between the two groups (the healthy controls: mean = 0.11 ± 0.052 mm, the children with GTCS: mean = 0.15 ± 0.09 mm). Nuisance signals (white matter, cerebrospinal fluid and six head motion parameters) were regressed out to correct for head motion and physiological noise.

### 2.4 Network construction and topological analysis

The brain network topological characterizations were analyzed using GRETNA (http://www.nitrc.org/projects/gretna/) (Wang et al., 2015). First, the brain was divided into 90 cortical and subcortical regions of interest based on an automated anatomical labeling atlas. Each region was regarded as a network node. Second, the mean time series in each region was extracted. For each participant, Pearson's *r* correlations of the mean time series between all pairs of nodes were calculated. Third, to normalize the variance in correlation values, all resulting correlation coefficients were transformed into *z*‐scores using Fisher's *z*‐transformation. The normalized correlation value of each pair was regarded as the network edges. Thereafter, a 90 × 90 correlation matrix was produced for each subject. A binary matrix was obtained according to a predefined threshold (see below for the threshold selection), where edges with positive correlation values were set to 1 and otherwise set to 0. Finally, different levels of network topological properties were performed. The global network graph metrics were assessed in terms of small‐world (small‐worldness (*σ*), clustering coefficients (*C*_p_), normalized clustering coefficient (*γ*), characteristic path length (*L*_p_), normalized characteristic path length (*λ*)), and network efficiency (local efficiency (*E*_loc_) and global efficiency (*E*_g_)). The regional characteristics, such as nodal efficiency, nodal local efficiency, and nodal shortest path, were also assessed.

### 2.5 Statistical analysis

Because there is currently no definitive way to select a single threshold, we thresholded each correlation matrix repeatedly over a wide range of sparsity values. The range of sparsity values was chosen here to allow small‐world network properties to be properly estimated. In the present study, the range of sparsity thresholds was from 0.1 to 0.5 with an interval of 0.01. At the lower bound of the range, the networks of both groups were not fragmented. For densities above 0.45, the graphs became increasingly random (*σ* < 1.5). The area under the curve (AUC) is a summarized scalar which can reflect the topological characterization of brain networks for each network metric. A previous study demonstrated that AUC was independent of single threshold selection and sensitive to topological alterations in brain disorders (Suo et al., 2015). Between‐group differences in the AUCs were analyzed using independent sample *t* test with age and gender as covariates (*p* < .05, false discovery rate (FDR) corrected). Between‐group differences were identified in the nodal metrics, and the significant results were visualized using the BrainNet Viewer software (Xia et al., 2013).

We used a network‐based statistic (NBS) approach for the functional connectivity networks to localize the specific connected components, which reflects the functional connections that differed between each pair of groups (Zalesky et al., 2010). The nodes that exhibited significant between‐group differences in at least one of the small‐world parameters were chosen for each subject. A subset of the connections matrix was generated. A set of suprathreshold links among all connected components was defined using the NBS method. The nonparametric permutation method was used to estimate the significance for each component (10,000 permutations). The threshold (*p* < .05, FDR correction) was adopted to address multiple comparisons in functional connectivity. Significant links between groups were also visualized using the BrainNet Viewer software.

Using the above process, significant between‐group differences in the network metrics were identified. We further calculated correlations between the metrics and the epilepsy duration in the patient group.

## 3 RESULTS

### 3.1 Group differences in global network metrics

**FIGURE 1:**
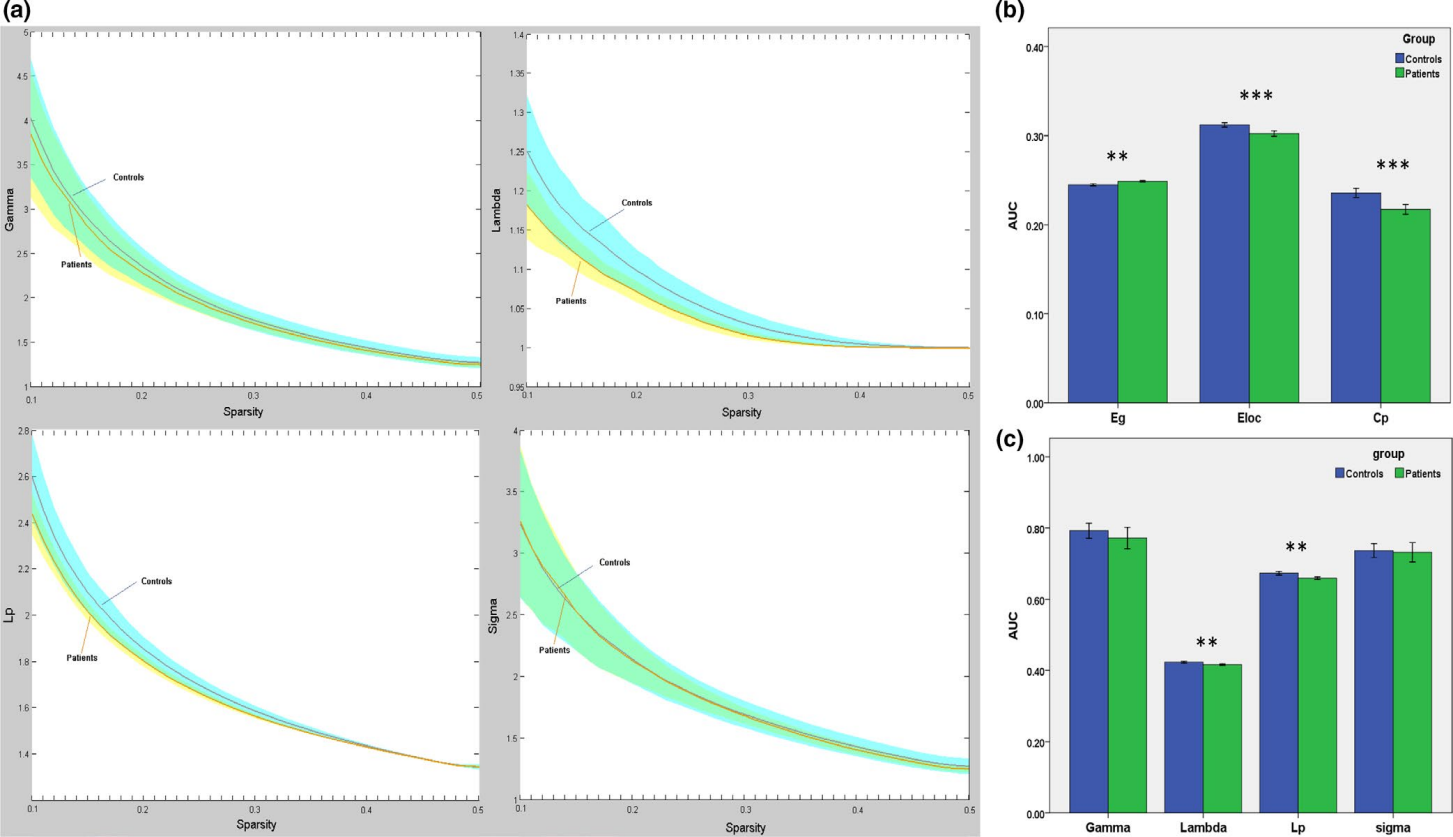
Group differences in global functional properties of brain topology between the patient and control group. (a) In the range of sparsity (0.1 ~ 0.5), the functional networks in both groups exhibited a small‐world property. (b) Bar plots of the global efficiency, local efficiency, and clustering coefficiency for the GTCS children and controls. (c) Bar plots of the gamma, lambda, *L*_p,_ and sigma for the GTCS children and controls. Asterisks (*) indicate a significant difference between the two groups. AUC, area under the curve; *E*_g_, global efficiency; *E*_loc_, local efficiency; *C*_p_, clustering coefficient; *L*_p_, characteristic path length

There no significant differences in age or gender distribution between the two groups. GTCS patients and HCs showed small‐world topology of the brain functional connectome: high *C*_p_ (*γ* > 1) and similar *L*p (*λ* ≈ 1). The result was unified using a metric called small‐worldness (*σ* > 1) (Figure 1a). Compared with the normal controls, the GTCS patients showed reduced *C*_p_ (*t* = 4.35, *p* = .000), *L*_p_ (*t* = 3.36, *p* = .002), *λ* (*t* = 3.46, *p* = .001), and *E*_loc_ (*t* = 4.38, *p* = .000) and increased *E*_g_ (*t* = 3.63, *p* = .001) (Figure 1b,c). No significant differences in *γ* or *σ* metrics were detected between the two groups.

### 3.2 Group differences in regional topological organization

**FIGURE 2:**
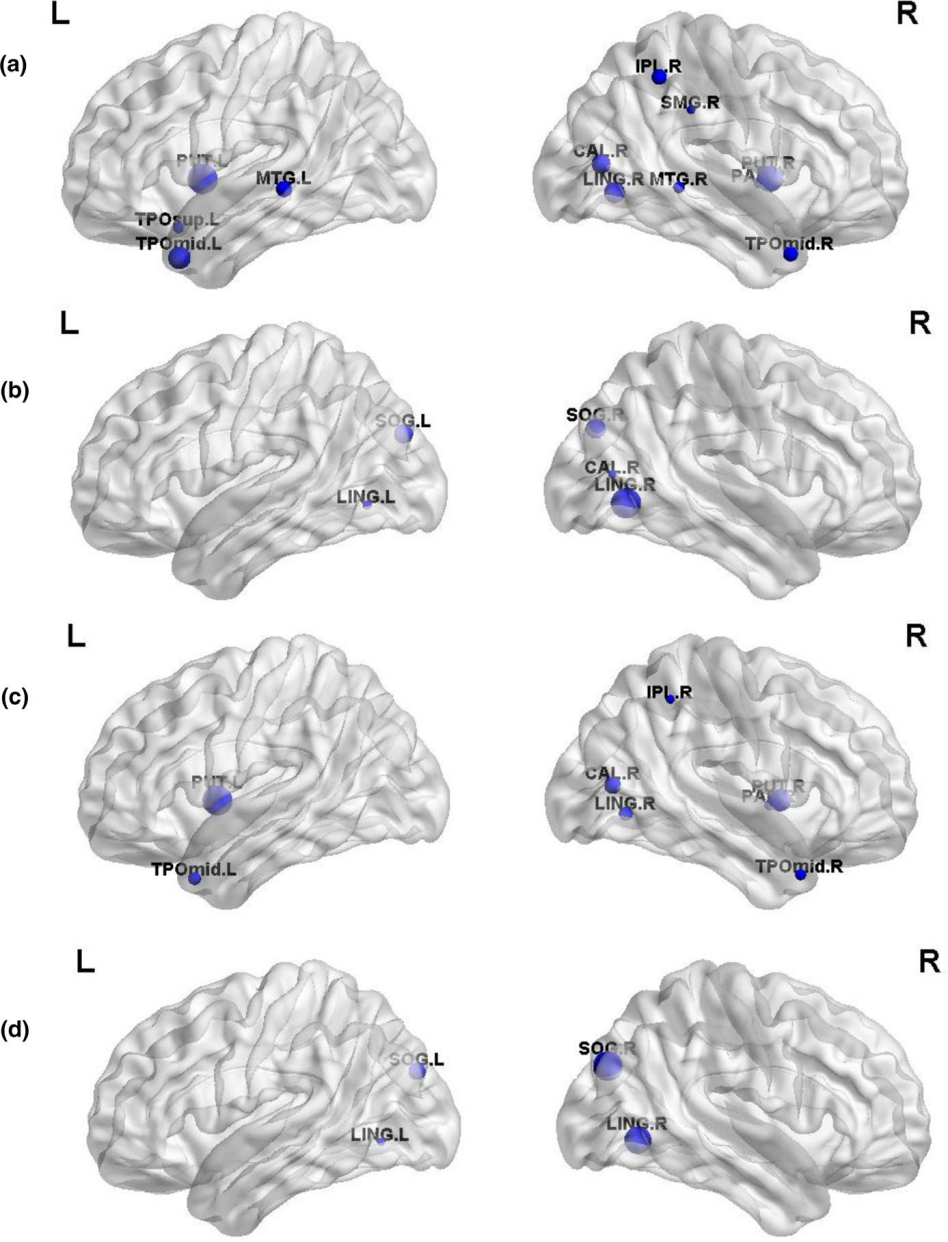
Brain regions showing significant intergroup differences of the each nodal topologic properties in brain functional networks in the children with GTCS compared with the controls. (a) decreased clustering coefficient, (b) increased nodal efficiency, (c) decreased nodal local efficiency, and (d) decreased nodal shortest path. The node sizes indicate the significance of between‐group differences in each nodal topologic properties. The abbreviations for the nodes are presented in Table 2

Brain regions with significant intergroup differences were identified (*p* < .05, FDR corrected, Table 2) and displayed the brain location in Figure 2. Compared with the controls, the children with GTCS showed decreased *C*p in the right calcarine, right lingual, right inferior parietal lobe (IPL), right supramarginal, right pallidum, left temporal pole of superior temporal gyrus (STG), bilateral putamen, bilateral middle temporal gyrus (MTG), and temporal pole of the MTG (Figure 2a). Increased nodal efficiency was found in bilateral lingual, bilateral superior occipital gyrus (SOG), and right calcarine sulcus (Figure 2b). For the patient group, significant decreases in nodal local efficiency were found in the right calcarine sulcus, right lingual, right IPL, right pallidum, bilateral putamen, and temporal pole of the MTG (Figure 2c). Significant decreases in nodal shortest path were found in the bilateral lingual and SOG (Figure 2d).

**TABLE 2:** Regions showing disrupted nodal topologic properties in GTCS children compared with the controls

Regions	Cluster coefficient (*t*/*p*)	Nodal efficiency (*t*/*p*)	Nodal local efficiency (*t*/*p*)	Nodal shortest path (*t*/*p*)
R CAL	−3.59/0.0009	3.61/0.00087	−3.7/0.00066	**–**
L LING	**–**	3.64/0.00078	**–**	−3.57/0.00096
R LING	−3.69/0.0007	4.4/0.00008	−3.54/0.0011	−4.1/0.00021
L SOG	**–**	3.99/0.0003	**–**	−3.82/0.00047
R SOG	**–**	4.04/0.00024	**–**	−4.18/0.00016
R IPL	−3.37/0.0017	**–**	−3.19/0.0028	**–**
R SMG	−2.99/0.0048	**–**	**–**	**–**
L PUT	−4.21/0.00014	**–**	−4.49/0.00006	**–**
R PUT	−4/0.00027	**–**	−4.03/0.00025	**–**
R PAL	−3.07/0.0039	**–**	−3.32/0.00197	**–**
L TPO of STG	−3.13/0.00326	**–**	**–**	**–**
L MTG	−3.42/0.00147	**–**	**–**	**–**
R MTG	−3.15/0.00313	**–**	**–**	**–**
L TPO of MTG	−3.78/0.00052	**–**	−3.47/0.0013	**–**
R TPO of MTG	−3.37/0.0017	**–**	−3.37/0.0017	**–**

Abbreviations: Bi, bilateral; CAL, calcarina sulcus; IPL, inferior parietal lobe; L, left hemisphere; LING, lingual gyrus; MTG, middle temporal gyrus; PAL, globus pallidus; PUT, putamen; R, right hemisphere; SMG, supramarginal gyrus; SOG, superior occipital gyrus; STG, superior temporal gyrus; TPO, temporal pole.

### 3.3 Group differences in functional connectivity

**FIGURE 3:**
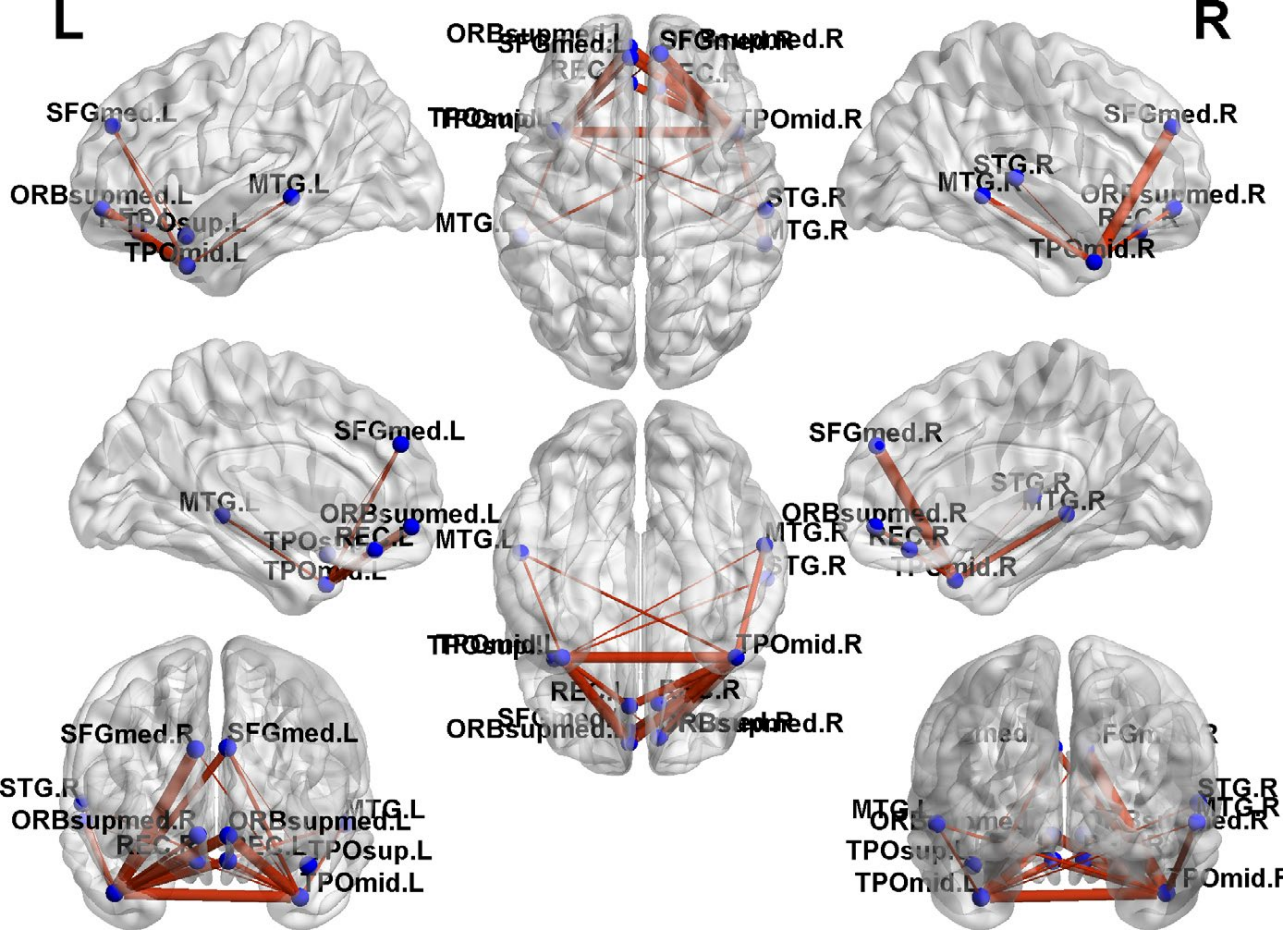
Connected networks that show decreased functional connections in the children with GTCS

**FIGURE 4:**
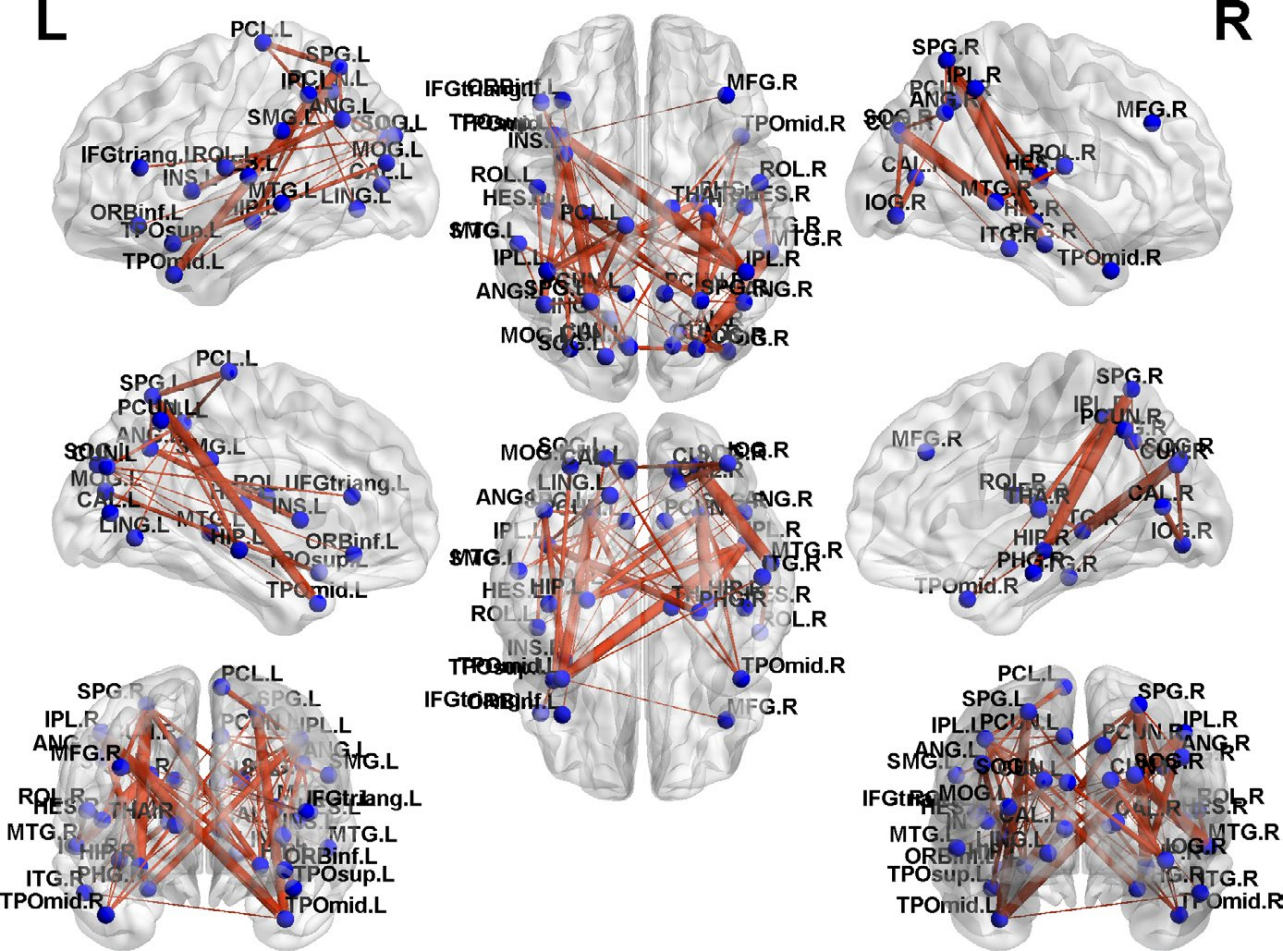
Connected networks that show increased functional connections in the children with GTCS

Based on the NBS analysis, a decreased functional connectivity network with 12 nodes and 18 connections was identified in GTCS patients, involving the bilateral medial superior frontal gyrus (SFG), temporal pole of STG and MTG (Figure 3). An increased functional connectivity network with 37 nodes and 54 connections was identified in the GTCS patients, involving the bilateral hippocampus, thalamus, occipital lobe, IPL, superior parietal lobe (SPL), angular, insula, temporal pole of STG, and MTG (Figure 4). The results were visualized using the BrainNet Viewer package.

### 3.4 Relationship between the network metrics and epilepsy duration

**FIGURE 5:**
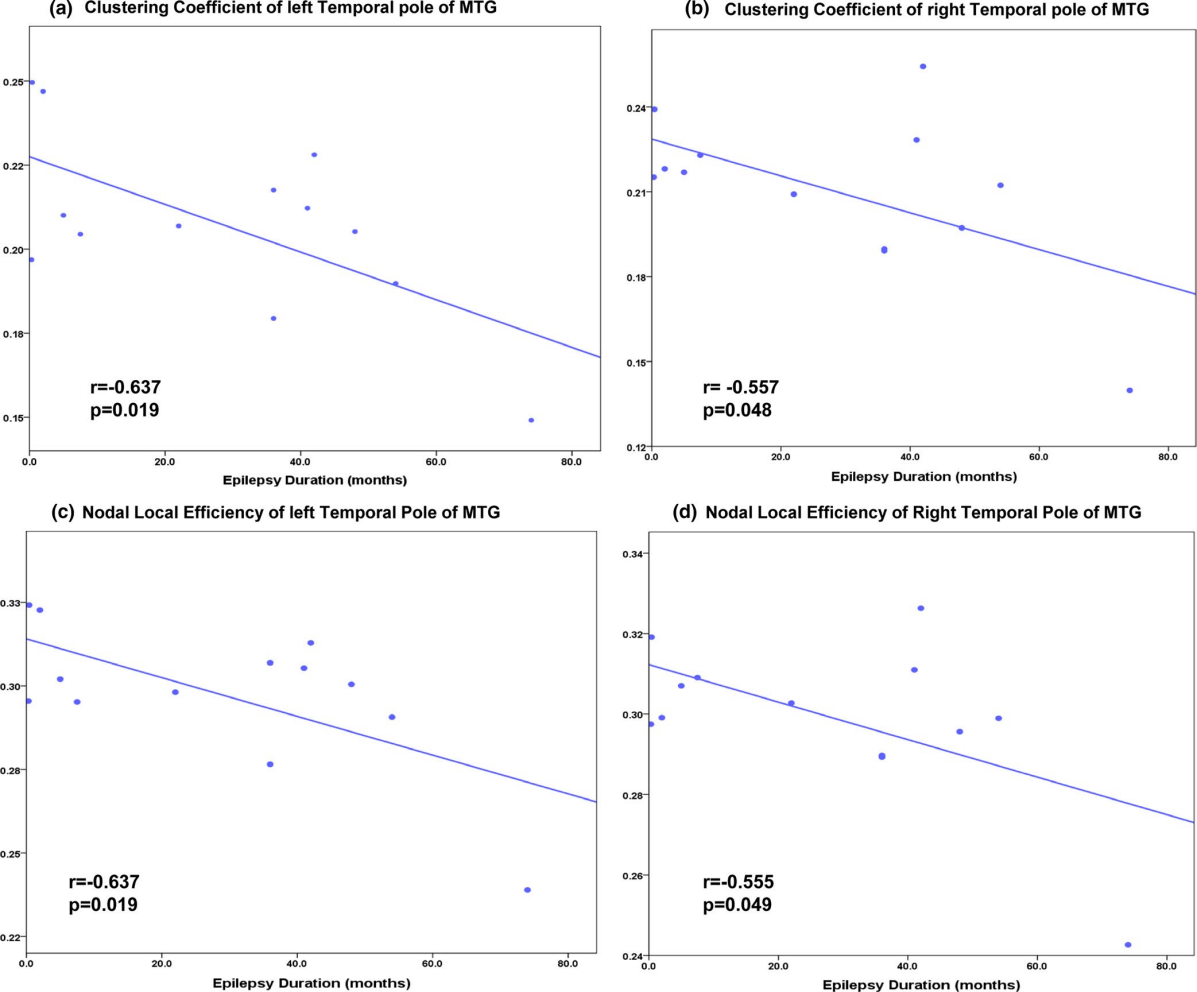
Correlation between the graph properties and the epilepsy duration. (a, b) Scatter plot showing significant negative correlation between epilepsy duration and clustering coefficient of bilateral temporal pole of MTG. (c, d) Scatter plot showing significant negative correlation between epilepsy duration and nodal local efficiency of bilateral temporal pole of MTG

In the patient group, significant correlations were found between the epilepsy duration and the clustering coefficient of the bilateral temporal pole of MTG (left: *r* = −0.637, *p* = .019, right: *r* = −0.557, *p* = .048, Figure 5a,b). The nodal local efficiency of the bilateral temporal pole of MTG also showed significant correlations with the epilepsy duration (left: *r* = −0.637, *p* = .019, right: *r* = −0.555, *p* = .049, Figure 5c,d).

## 4 DISCUSSION

In the present study, we used a graph‐based theoretical approach to investigate the topological alterations in functional brain connectomes in children with GTCS. Although the patients exhibited efficient small‐world properties of their functional networks that were similar to the normal controls, they also exhibited a significant decrease in clustering coefficient and nodal local efficiency in the bilateral putamen and temporal pole of MTG. Furthermore, GTCS children exhibited decreased network connectivity between regions, including the bilateral medial SFG, temporal pole of STG and MTG. Increased network connectivity was also found among regions, primarily the hippocampus, thalamus, occipital lobe, IPL, SPL, angular, insula, and temporal pole of STG and MTG. The clustering coefficient and nodal local efficiency in bilateral temporal pole of MTG were negatively correlated with epilepsy duration in children with GTCS. Together, our findings indicated that the brain network architecture and communities of the children with GTCS were changed at global and nodal levels. Our results provided preliminary evidence that the topological organization of function networks was disrupted in children with GTCS. Abnormal functional network properties in children with GTCS may help us to detect possible topological markers in epilepsy syndrome.

### 4.1 Altered global properties in the functional networks of children with GTCS

The human brain is a complex neural network that exhibits a balance between global integration and local specialization (Van Den Heuvel & Pol, 2010). In the present study, the graph theory method was used to map the topological properties of whole‐brain functional networks. Both groups showed small‐world properties (e.g., a lower *L*_p_ and higher *C*_p_, in Figure 1) of the functional networks. This result suggests that the networks of both groups showed high efficiency in information processing and transfer (Sporns & Honey, 2006). Demonstration of a small‐world property of the human brain network is the usual finding in healthy and disease research (Bernhardt et al., 2015; Fornito et al., 2013; Stam, 2014). Previous neuroimaging studies in epilepsy also found that patients with idiopathic generalized epilepsy or temporal lobe epilepsy demonstrated a small‐world property of the functional and structural networks (Bernhardt et al., 2011; Liao et al., 2010; Zhang et al., 2011). Therefore, the global graph results of the present study indicated that children with GTCS retained the small‐world properties of the functional network, which was consistent with the findings of adults with GTCS (Li et al., 2016; Liao et al., 2013).

Although the small‐world properties of the functional networks were preserved in children with GTCS, the detailed alterations in overall topology were different from adults with GTCS (Zhang et al., 2011). This previous study demonstrated a significant decrease in *σ* and *γ* in adults with GTCS. In the present study, although the children with GTCS also showed a decreased *σ* and *γ*, these changes did not reach statistical significance. One possible reason for the difference between the children and adults may be different development effects. Childhood is a specific period with fast development of the brain. Development via the growth process progressively fine‐tunes the configuration of the nodes and edges. For children with GTCS, the topology changes in their large‐scale network were the combined effects of epilepsy and development. Therefore, the overall topology in children with GTCS was not fully consistent with that in adults with GTCS.

The local efficiency and clustering coefficient in children with GTCS were significantly decreased compared with the normal controls in some regions. Simultaneous decreases in both *E*_loc_ and the *C*_p_ indicated that the network architecture of information transfer and processing across the brain was changed in children with GTCS. A random network has a more global character with a lower *C*_p_ and a much shorter path length than the regular network (Van Den Heuvel & Pol, 2010). A small‐world network is characterized by a high *C*_p_ and a short path length (Watts & Strogatz, 1998). In the present study, children with GTCS showed significant decreases in nodal shortest path and *C*_p_ compared with the normal controls. These results may indicate that the brain network architecture of children with GTCS had a tendency to shift from a small‐world organization to a random network. This inference is consistent with a previous graph theory study that the interictal network topology of individuals with epilepsy seemed to have a random shift from small‐world organization (Li et al., 2016). Therefore, the findings of the present study suggest that children with GTCS showed a topological reorganization of the functional networks. Combining with the previous graph theory studies on children with epilepsy (Li et al., 2020; Qiu et al., 2017), the present study indicates that long‐term epilepsy history disrupts children's brain topology. The overall trend of brain topology in children with epilepsy would different from the adult with epilepsy. The research in children with epilepsy should deserve more attention as that the results in children may be the combination of epilepsy and development. This view can give some advises on the understanding of brain topology in children with other siezure types.

### 4.2 Distributed regions with altered topological organization in children with GTCS

In this study, we also observed several brain regions with a reduced *C*_p_ and nodal local efficiency in children with GTCS, primarily in the right calcarine sulcus, right lingual, right IPL, right pallidum, bilateral putamen, MTG, and temporal pole of MTG. Previous studies have shown that the above regions were the key nodes of brain networks in adults with GTCS and exhibited functional abnormalities (Li et al., 2016; Song et al., 2011; Zhang et al., 2011). Most of these regions belonged to a default mode network or movement‐related network. The present study found that the right IPL, bilateral MTG and temporal pole of MTG showed significant decreases in the *C*_p_ and nodal local efficiency in children with GTCS. These regions are the main components of the DMN. Previous studies in patients with GTCS reported that these regions were highly related to functional integrations within the DMN (Blumenfeld et al., 2009; Liao et al., 2013; Liu et al., 2017; Song et al., 2011). The DMN showed more significantly altered connectivity than other resting‐state networks in the patients with idiopathic generalized epilepsies (Parsons et al., 2020). This network and associated regions of interest were frequently deactivated at rest among all idiopathic generalized epilepsy subtypes compared to the normal controls. Our recent studies in children with GTCS also found that the temporal regions of the DMN in the patient group showed abnormal brain activity and local coherence (Wang et al., 2018). Graph theory analysis on the GM structural covariance network found that children with GTCS showed significant alterations of the nodal betweenness in the right thalamus, bilateral temporal pole, and some regions of DMN (Li et al., 2020). In the present study, significant decreases of *C*_p_ and nodal local efficiency in children with GTCS support the viewpoint that chronic epilepsy may disrupt the regional integration of their functional networks. The present results are consistent with these previous studies and indicate that chronic epilepsy caused functional reorganization in children with GTCS. The putamen is a round structure that is located in the center of the brain. The putamen region plays a role in movement regulation and learning. Previous neuroimaging studies revealed that adults and children with idiopathic generalized epilepsy demonstrated increased brain activity in the putamen, insula, thalamus, and cerebellum (Li et al., 2016; Luo et al., 2012; Wang et al., 2014). Structural neuroimaging studies in patients with GTCS also demonstrated a significant decrease in gray matter volume in the putamen (Ciumas & Savic, 2006). These studies consistently pointed out that the putamen was an epilepsy‐related area. Nodal topology alteration in the bilateral putamen in children with GTCS may underlie the uncontrolled behavioral problems, such as involuntary movements (Mink, 2003). In the present study, significant decrease in the *C*_p_ and nodal local efficiency in these regions suggest that the changed topological properties were the result of chronic epilepsy in the children.

GTCS is characterized by generalized spike‐wave discharge involving the bilateral hemispheres during seizures. Recent studies of simultaneous electroencephalography and fMRI in idiopathic generalized epilepsy have focused on the role of bilateral distributed cortical and subcortical networks as the sources of seizures (Bai et al., 2010). In the present study, we observed a significant decrease in *C*_p_ and nodal local efficiency in the bilateral putamen, MTG and temporal pole of MTG of children with GTCS. The regions are different from the unilateral distribution of significant nodal characteristic alterations reported in adults with GTCS (Zhang et al., 2011). A bilateral alteration of nodal characteristics in adults with GTCS was only found in the anterior cingulate gyrus. Therefore, the effect of GTCS on the brain is similar, but not fully consistent between adults and children. Bilateral changes in nodal characteristics in the putamen and MTG may be a specific imaging expression in children with GTCS. This view needs further investigation in future studies. Significant alterations of nodal topological characteristics were also found in some regions of the right hemisphere, but not the left hemisphere, of children with GTCS. This result was consistent with the asymmetry spatial distribution of altered nodes discovered in adults with GTCS in a previous investigation (Zhang et al., 2011). This finding was also confirmed in other epilepsy studies, which demonstrated asymmetric spatial distribution in patients with idiopathic generalized epilepsy (Casaubon et al., 2003; Walser et al., 2009). The underlying mechanism for children with GTCS showing symmetrically change in some regions and asymmetric change in other regions is still unclear, This phenomenon may be specific for children with GTCS which need further investigation.

### 4.3 Functional connectivity alterations in children with GTCS

In the present study, children with GTCS exhibited a significant decrease of brain network connectivity, primarily in the pathways connecting the bilateral SFG, temporal pole of STG and MTG. The functional connectivity between the corresponding nodes in the patients was significantly decreased compared with the normal controls, especially the connectivity between the bilateral temporal pole of MTG and the connectivity between temporal lobe and SFG. The significantly decreased connectivity between the bilateral temporal pole is consistent with a previous neuroimaging study that showed aberrant changes in the interhemispheric functional connectivity between the bilateral temporal pole and bilateral olfactory cortex in adults with GTCS (Ji et al., 2014). A significant decrease in functional connectivity between the temporal lobe and SFG may be explained by the reduction in functional integrations of the DMN in children with GTCS. Studies in adults with GTCS found that chronic epilepsy impaired the intrinsic brain activity of the DMN (Liao et al., 2013; Parsons et al., 2020; Song et al., 2011; Zhang et al., 2011). Because the temporal lobe and SFG belong to the DMN, decreased connectivity between these regions suggests that chronic epilepsy impairs the integration of the resting‐state connectivity pattern within the DMN. This inference was confirmed by the nodal topology results, which showed that children with GTCS had a decreased clustering coefficient in temporal lobe.

For children with GTCS, network components with increased functional connections were primarily involved in the pathways connecting nodes such as the hippocampus, thalamus, occipital lobe, IPL, SPL, angular, insula, and temporal pole. The thalamus likely serves as a source of spike and slow‐wave discharge activity (Wang et al., 2011). The thalamus also plays an important role in the initiation, propagation and inhibition of epileptic activity. The cortical–thalamic–subcortical circuits are specialized to exert thalamic control during cognitive processing (Zhang et al., 2015). Neuroimaging studies in adults and children with GTCS also demonstrated abnormalities in brain structure and function in the thalamus (Huang et al., 2011; Wang et al., 2012, 2019). Patients with GTCS showed a marked imbalance in both components of the cortico–thalamic system compared with the healthy controls (Wang et al., 2019). More restricted thalamic connectivity may contribute to the hyperexcitability of the thalamus in patients with GTCS. The children with GTCS in the present study showed increased functional connectivity between the right thalamus and bilateral IPL. This result confirmed the view that chronic epilepsy induces functional abnormalities of the thalamus. Bilateral temporal pole and hippocampus also showed significant increases of functional connectivity to the bilateral SPL and IPL in the patient group. The temporal pole and hippocampus are the main regions of the DMN, and the parietal regions are key components in the frontoparietal network. Increased connectivity between the DMN and frontoparietal network suggests that the disrupted neural activity within DMN regions were compensated by the increased internetwork connection to transmit functional information. Enhanced internetwork connections and decreased intranetwork connections indicated that the architecture of the brain networks was reconfigured in children with GTCS.

### 4.4 Clinical relevance of network alterations in children with GTCS

Notably, the *C*_p_ and nodal local efficiency of the bilateral temporal pole of MTG were significantly correlated with epilepsy duration in children with GTCS. The negative correlations between brain topological properties and clinical history are consistent with some previous neuroimaging studies (Li et al., 2016; Zhang et al., 2011). The relationship result in the present study indicated that children with a longer history of epilepsy would demonstrate a greater decrease in nodal topological properties. This result suggests that chronic epilepsy disrupts the topological properties of these regions. Significant correlations were detected in the bilateral temporal pole but not the other nodes, which may relate to its structural connection with the thalamus. A previous study showed that the thalamus played a crucial role in the secondary generalization of epileptic seizures (Norden & Blumenfeld, 2002). During epileptic seizures, aberrant brain activity was generated in the bilateral temporal lobes via thalamocortical projection (Bernhardt et al., 2009). The brain functional connectivity results of the present study also support this explanation. Children with GTCS showed increased functional connections primarily involving the pathways connecting the thalamus and temporal pole. In the domain of brain topology, epilepsy may induce significant changes in the topological properties in the bilateral temporal poles of the MTG (Li et al., 2020). Because significant discoveries in the bilateral temporal poles of MTG were detected in all domains of our analyses, such as regional properties, functional connectivity, and correlation, the topological properties in these regions may be considered the potential connectome‐based biomarker for reflecting chronic damaging effects of GTCS in children. Future studies should be performed to test the differences between adults and children with GTCS.

### 4.5 Limitations

Several limitations must be addressed in the present study. First, the sample size of the patient group was relatively small. Future studies with larger sample sizes may provide further insights into the issue. Second, we did not collect the cognitive function of the participants in the present study. A previous study showed that specific cognitive functions were associated with brain network topological characteristics (Wen et al., 2011). Further studies should collect cognitive performance to investigate whether alterations of network topologies in children with GTCS reflect cognitive ability. Third, some younger children were sedated with 10% chloral hydrate during the image collecting. The use of sedative on the younger children may affect the connectivity measures. Finally, the entire collection time of our fMRI data was relatively short compared with the previous epilepsy references. The intention of the design was that some children cannot maintain a good condition during a long scanning time, unlike adults. However, the short scanning time may reduce the stability of our analysis results. This factor should be considered in future studies.

## 5 CONCLUSIONS

Using the graph theory method, we investigated the reorganization of the complex brain network in children with GTCS. A small‐world topology of the functional connectivity networks was observed in children with GTCS and the normal controls. The architecture of their functional brain network showed a tendency toward a random network. Significant decreases of nodal local efficiency and clustering coefficient were found in children with GTCS in a few key nodes. The connections within the DMN were decreased significantly, and the internetwork connections (DMN‐frontoparietal network and the thalamic–cortical network) were increased significantly. The changed topological properties may result from the effect of chronic epilepsy in children with GTCS. The optimal topological organization of the functional connectivity network was disrupted. Notably, clustering coefficient and nodal local efficiency of the bilateral temporal pole of MTG showed negative correlations with epilepsy duration in the patient group. The bilateral temporal pole may play a crucial role in reflecting the effects of chronic epilepsy in children with GTCS. This result is novel and important compared with the unilateral region characteristics reported in GTCS adults in previous neuroimaging studies. Further studies should be performed to confirm the result in the future. Overall, the present study preliminarily demonstrated a disrupted topological organization of the functional network in children with GTCS. The use of the graph theory method for neuroimaging data may serve as a possible topological marker in the diagnostics of epilepsy syndromes in children.

## CONFLICT OF INTEREST

None declared.

## AUTHOR CONTRIBUTIONS

YL and QC conceived and designed the experiments. YL performed the experiments. YL analyzed image data and sorted the results. QC and WH responsible for patient management and conceptualized the study. YL wrote and reviewed the paper.

### Peer Review

The peer review history for this article is available at https://publons.com/publon/10.1002/brb3.1890.

## Data Availability

The data that support the findings of this study are available from the corresponding author upon reasonable request.
